# Sleep Quality as a Mediator of Internet Gaming Disorder and Executive Dysfunction in Adolescents: Cross-Sectional Questionnaire Study

**DOI:** 10.2196/68571

**Published:** 2025-07-09

**Authors:** Michoel L Moshel, Wayne Warburton, Rainer Thomasius, Kerstin Paschke

**Affiliations:** 1 School of Psychological Sciences, Macquarie University, Sydney, Australia; 2 German Center for Addiction Research in Childhood and Adolescence, University Medical Center Hamburg-Eppendorf, Building W29 Martinistr 52, Hamburg, 20246, Germany, 49 40741059307

**Keywords:** internet gaming disorder, executive dysfunction, sleep quality, adolescents, digital media behaviors, sleep impairment

## Abstract

**Background:**

Internet gaming disorder (IGD) has been associated with impairments in executive functioning, particularly inattention and impulsivity. Sleep quality has separately been linked to both gaming behavior and cognitive performance, yet its role as a mediating factor in this relationship is underexplored.

**Objective:**

This study aimed to determine whether sleep quality mediates the relationship between IGD symptoms and executive dysfunction in adolescents, specifically focusing on the domains of inattention and hyperactivity or impulsivity. A reverse mediation model was also tested to explore the bidirectional nature of these relationships.

**Methods:**

A representative sample of 1000 adolescents (539/1000, 53.9% males), aged between 12 and 17 years (mean 14.52, SD 1.64), completed validated self-report measures of IGD symptoms, executive dysfunction, and sleep quality. Structural equation modeling was used to test direct and indirect effects with age and gender included as covariates.

**Results:**

Of the sample, 2.4% (24/1000) met criteria for IGD (875/1000, 87.5% males), and 22.6% (226/1000) met criteria for chronic sleep reduction. Among those with IGD, 54.2% (542/1000) also experienced chronic sleep reduction. In model A (IGD → Sleep → Executive Dysfunction), IGD symptoms were associated with poorer sleep quality (*a*=0.32, 95% CI 0.19-0.44), which in turn were associated with greater executive dysfunction (*b*=0.05, 95% CI 0.01-0.10). The indirect effect was significant (*a×b*=0.02, 95% CI 0.01-0.04), and sleep quality was a partial mediator. In the reverse model (model B), executive dysfunction was associated with poorer sleep quality (*a*=0.15, 95% CI 0.06-0.25), which subsequently was associated with higher IGD symptoms (*b*=0.11, 95% CI 0.07-0.16); indirect effect *a×b*=0.02, 95% CI 0.01-0.04. Simple slope analysis showed that IGD symptoms were associated only with executive dysfunction at average or poor levels of sleep quality. At higher levels of sleep quality, this relationship was no longer significant.

**Conclusions:**

The results of this study suggest that sleep quality may be an important intermediary mechanism by which IGD might contribute to executive dysfunction and provide a basis for the development and implementation of strategies that target sleep issues in IGD. Prospective longitudinal research is needed to examine the directionality of the relationships between IGD, sleep quality, and executive dysfunction longitudinally.

## Introduction

Over the past few decades, gaming and screen use have surged in popularity due to technological advancements and the increasing availability and accessibility of these technologies [1]. For some individuals, this rise in gaming behavior has led to problematic relationships with gaming and the internet. The inclusion of gaming disorder (GD) in the *ICD-11* (*International Classification of Diseases, Eleventh Revision*) by the World Health Organization highlights the growing recognition of these issues, describing it as a pattern of persistent or recurrent gaming behavior that takes precedence over other life interests and activities, resulting in significant impairment or distress [2]. Similarly, the *DSM-V-TR* (*Diagnostic and Statistical Manual of Mental Disorders, Fifth Edition, Text Revision*) acknowledges internet gaming disorder (IGD) as a condition warranting further study [3]. While both IGD and GD describe problematic gaming behaviors, IGD follows an addiction-based model with a focus on psychological harm, whereas GD emphasizes functional impairment [4]. Additionally, GD is less stable over time and more associated with psychiatric comorbidities [5].

A meta-analysis exploring rates of IGD indicated a global pooled prevalence of 4.6% [6], with other studies estimating prevalence rates of 1.16%‐3.5% in German adolescents [7,8], 3.6%‐9.4% in North American samples [9,10], and 2.8%‐3.1% in Australian teenagers elevated under the COVID-19 pandemic [11-13]. This prevalence is concerning, given the particular susceptibility of adolescents to developing IGD [14-16], along with the neurological, psychological, and social consequences associated with its development [17-20]. Globally, IGD treatment centers have seen a steady increase in patient referrals with common presenting issues including family conflict, social isolation, and gaming-related interference with other activities [21].

Although the research field still lacks a clear consensus on the precise operationalization of IGD and its diagnostic parameters [22-25], evidence increasingly indicates that IGD and other disordered screen use behaviors are associated with cognitive impairments in clinical populations [26-29]. A recent meta-analysis on neuropsychological performance in individuals with problematic gaming behaviors and broader disordered screen use found decreased cognitive performance, with small to medium effect sizes compared with healthy controls [26]. The most affected cognitive domains were attention and executive functioning [26].

This paper focuses on executive functioning, specifically on 2 key facets: attentional control (inattention) and behavioral inhibition (hyperactivity or impulsivity). While executive functioning also encompasses working memory, cognitive flexibility, and higher-order processes such as decision-making and problem-solving [30,31], this study specifically examines the regulatory control of attention and behavior as core components of executive dysfunction in IGD. Moreover, these subdomains align closely with attention-deficit/hyperactivity disorder (ADHD) symptomology, a condition widely regarded as a disorder of executive dysfunction [32,33], and one that shows strong comorbidity with IGD [34-37]. Specifically, higher GD symptom severity is related to higher symptoms across the hyperactivity or impulsivity and inattention subdomains [38]. This overlap highlights the nature of executive dysfunction across both disorders, providing a theoretically and clinically grounded rationale for focusing on these executive function domains in this study.

Impairments in healthy executive functioning, a crucial part of brain development during childhood and adolescence [39], can have far-reaching implications for socioeconomic status, including academic success, school dropout rates, overall health, and quality of life [40-43]. Adolescents with IGD exhibit neurobiological changes, such as reduced orbitofrontal cortex thickness, a structural abnormality also seen in substance use disorders [44], as well as abnormal glucose metabolism and white matter fiber consistency in the orbitofrontal regions [45]. Indeed, Ioannidis et al [46] characterize cognitive dysfunction as part of the pathophysiology of problematic internet use, including problematic gaming, particularly highlighting the existence of underlying frontostriatal dysfunction. In a systematic review, Schettler et al [47] examined problematic gaming—a term encompassing both IGD and GD—and found that adolescents exhibiting problematic gaming behaviors showed greater cognitive-affective imbalance than age-matched controls. This imbalance was marked by alterations in brain regions associated with identity formation, social cognition, personality formation, and mentalizing—key processes during this developmental period. Although establishing a causal mechanism between problematic gaming behaviors and executive dysfunction is challenging [48,49], it is possible that secondary effects resulting from gaming and executive dysfunction, such as sleep, may be mediating this relationship.

Indeed, previous studies have highlighted the crucial role that sleep plays in cognitive functioning, particularly executive control, as well as in the development of IGD. For instance, addicted gamers reported significantly higher rates of daytime sleepiness and sleep deprivation [50], with the use of screens more generally being associated with symptoms of insomnia [51]. Sleep quality was shown to be lowest in children who use internet gaming for more than 6 hours a day and highest for those using only 1 to 2 hours a day [52]. A meta-analysis found that problematic gamers reported more adverse sleep status, including sleep duration, sleep quality, sleep problems, and daytime sleepiness, than nonproblematic gamers [53]. In a longitudinal study, Barlett et al [54] found that sleep mediated the relationship between media exposure (measured by screen time) and attentional problems in children, supporting the “displacement hypothesis,” which posits that screen time displaces time spent on more beneficial activities such as sleep [55].

Regarding executive functioning, the effects of sleep deprivation and reduced sleep quality have also been well studied. Various meta-analytic findings show a significantly negative effect of sleep restriction on executive functioning, sustained attention, and long-term memory, with the magnitude of the effect shown to increase with age [56-58]. Sleep problems may also interact with ADHD symptoms via reciprocal causation, possibly sharing a common neurological etiology [59]. Quality of sleep may be adversely impacted in ADHD with increased sleep-onset latency, shorter sleep time, sleep-disordered breathing, and nocturnal motricity [60]. Poorer sleep quantity and quality in adolescents is associated with weaker attentiveness and poorer responses on tasks of executive functioning [61]. Given these decrements in executive function, Anderson et al [62] recommend that pediatricians and public health officials consider sleep quality and deprivation as an important contributor regarding adolescent functioning.

With this recommendation in mind, it currently remains unclear whether sleep quality mediates the relationship between IGD and executive function, particularly in core components of inattention and hyperactivity/impulsivity. Additionally, there is uncertainty regarding the pathway of these effects: whether executive dysfunction or IGD leads to reduced sleep quality in the same way and, conversely, which condition is more considerably impacted by poor sleep quality. Identifying sleep quality as a mediator would allow researchers and clinicians to focus on it as a key factor linking IGD and executive dysfunction, making it a target for intervention. Early identification of sleep problems in individuals at risk for IGD or executive dysfunction could serve as a preventive measure and positively influence patient prognosis [63]. This approach suggests that, rather than solely focusing on reducing gaming or directly managing executive dysfunction, interventions could include strategies to improve sleep, which could incrementally add to better overall outcomes. This is especially important given the disproportionate risk adolescents face in developing IGD, where early intervention in the developing brain is particularly crucial [64].

To the authors’ knowledge, no studies have examined sleep quality as a potential mediator between the severity of IGD and executive dysfunction. Therefore, based on prior theoretical foundations, we hypothesize that sleep quality will mediate IGD and executive dysfunction, focusing on the subdomains of inattention and hyperactivity/impulsivity. As this is a cross-sectional study and given the support for a bidirectional model of executive dysfunction and IGD symptoms as well as the uncertainty regarding pathway effects, the reverse mediational process will also be examined. The results of this study may inform future longitudinal and prospective research exploring these outcomes.

## Methods

### Ethical Considerations

This study is part of a large ongoing study on adolescent media usage [65-67] for which ethical approval (no. LPEK-0307) has been obtained by the local psychological ethics commission at the Center for Psychosocial Medicine of the University Medical Center Hamburg-Eppendorf. Verbal informed consent was obtained from both parents and adolescents prior to their participation in the study. Participants were informed of their right to withdraw from the study at any time without any consequences. Compensation was offered for participation in the form of charity vouchers. The authors assert that all procedures contributing to this work comply with the ethical standards of the relevant national and institutional committees on human experimentation and with the Declaration of Helsinki of 1975, as revised in 2008.

### Participants

Data were collected by Forsa, a market and opinion research institute, using computer-assisted telephone interviews (forsa.omniTel). Participants were children and adolescents aged 12-17 years, selected through a multistage systematic random sampling process based on the German *Arbeitskreis Markt- und Sozialforschungsinstitute e.V*. (ADM; a business association representing private sector market and social research agencies) telephone master sample. Sampling occurred in 2 stages: first, geographically distributed sampling points were identified across Germany; second, households within these points were selected via a random-route method, with 1 eligible adolescent randomly chosen per household. A total of 2075 households were contacted, and after obtaining verbal consent from parents and participants, the final participation rate was 48.2%, yielding a sample of 1000 participants. The dual-frame sampling method (landline and mobile numbers) ensured broad coverage of the population. The sample was stratified by age, gender, and region to align with national demographic distributions based on microcensus data from the Federal Statistical Office of Germany. Data were also collected on educational and occupational status (school student, apprentice, and voluntary service), school attainment (desired or achieved school-leaving certificate), and recent attendance patterns (absences from school, apprenticeship, or work within the preceding 4 weeks). A rolling sample approach was used, meaning that there was no fixed gross sample; instead, previously processed numbers were continuously replaced with new numbers following a systematic call plan. Representativity using this approach has been established in prior research [68-71]. Participants were informed that their participation was voluntary, the data were collected anonymously, and would not be passed on to third parties. Due to the nature of telephone interviews, there were minimal missing responses, which were recorded as NA or “don’t know” responses. A comprehensive description of the data collection methods and demographic characteristics can be found elsewhere [67].

### Measures

To evaluate IGD symptoms as defined by *DSM-V*, we used the well-established Internet Gaming Disorder Scale (IGDS). This scale is a single-factor, polythetic tool comprising 9 questions with a binary response format (no/yes), with higher scores reflecting greater risk for IGD. It has been frequently used with German adolescent samples, showing good psychometric properties [67]. For the purposes of the descriptive results, the cutoff indicating disordered gamers is a score of at least 5 [72]. In this sample, the Cronbach α score was 0.58. Potential reasons for this low score are provided in the “Strengths and Limitations” section in the “Discussion” section.

Sleep quality was assessed by administering the 9-item, validated Sleep Reduction Screening Questionnaire (SRSQ) [73]. The SRSQ measures chronic sleep reduction including its associated consequences, affecting everyday life across 9 questions with a 3-step ordinal scale. The SRSQ has been validated and used in various populations, including German adolescents, with good psychometric properties [65,73]. Higher scores indicated more pronounced indications of poor sleep quality, with scores above 17.3 indicating chronic sleep reduction according to the Youden criterion reported elsewhere [65]. In this sample, the Cronbach α score was 0.71.

The Strengths and Difficulties Questionnaire (SDQ) is a widely used screening tool for assessing behavioral and emotional difficulties in children and adolescents as well as a sensitive screener for ADHD-combined subtype [74,75]. In this study, we used the hyperactivity-inattention subscale of the SDQ to assess 2 core components of executive functioning: attentional control (inattention) and behavioral inhibition (hyperactivity or impulsivity). These domains are commonly impaired in individuals with IGD and are also core symptoms of ADHD, which is itself widely recognized as a disorder of executive dysfunction [32,33]. Executive dysfunction, in the context of this study, is operationally defined as difficulty regulating attention and behavior, as measured by self-reported inattentiveness and hyperactivity. While this subscale does not capture the full spectrum of executive functioning, it targets 2 key subdomains most relevant to an IGD population. Responses were rated on a 3-point Likert scale: “not applicable,” “partially applicable,” and “clearly applicable.” Higher scores indicate greater executive functioning problems. This subscale has been used in child psychiatric diagnostic settings and demonstrates satisfactory to good psychometric properties [76-78]. In this sample, the Cronbach α score was 0.62.

### Data Analysis

**Figure 1.**
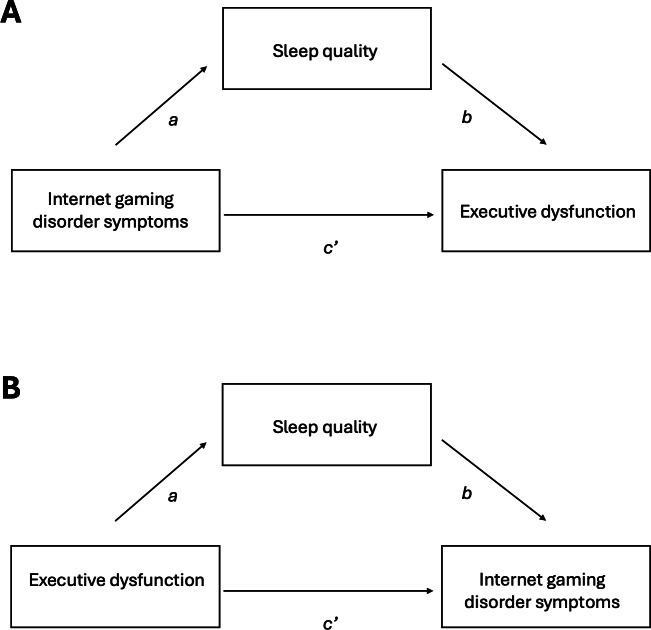
Hypothesized mediational models. In the first hypothesized mediation model (**A**), *a* represents the impact of Internet gaming disorder symptoms on sleep quality, while *b* represents the effect of sleep quality on executive functioning. The direct effect of Internet gaming disorder symptoms on executive functioning is denoted as *c′*. In model (**B**), the direction between Internet gaming disorder and executive functioning is reversed.

We followed recommendations for hypothesizing and constructing mediation models and interpreting effects [79-82]. To examine whether sleep quality mediates the effect of IGD symptoms on executive dysfunction, we used structural equation modeling and a nonparametric bootstrap method with bias-corrected CIs. Figure 1 shows the path diagram of the mediation models. As shown in Figure 1A, we tested the following effects: (1) effect of IGD symptoms on sleep quality (path *a*), (2) effect of sleep quality on executive function (path *b*), (3) indirect effect of IGD symptoms on executive functioning through sleep quality (path *a×b*), (4) direct effect of IGD symptoms on executive functioning, controlling for sleep quality (path *c′*), and (5) total effect, representing the overall effect of IGD symptoms on executive functioning (path *a×b+c*). An additional mediation model was run, reversing the predictor and outcome variables (Figure 1B).

Bootstrapping was used because it does not require the indirect effect (*a×b*) to be normally distributed, making it the preferred method to determine whether the indirect effect is different from zero [82,83]. A significant indirect effect suggests at least partial mediation, meaning that sleep quality explains part of the relationship between IGD symptoms and executive functioning. If the direct effect is nonsignificant in the presence of a significant indirect effect, this indicates that the effect of the predictor on the outcome variable occurs entirely through mediating variables (ie, sleep quality). If the direct effect remains significant, sleep quality only partially explains this relationship (partial mediation).

Software R (version 4.2.2; R Foundation for Statistical Computing) was used for data cleaning and analysis. To account for missing data, multiple imputation was used for each model. We used 60 imputations as recommended by Zhang and Wang [83] for the 21.6% missing data of 1 variable (IGDS) considered to be missing at random. The multiple imputation and mediation analyses were conducted using the *bmem* package (version 2.1; Comprehensive R Archive Network), which imputes missing data and runs the structural equation modeling in each of the 1000 bootstrap samples that were used in the analysis. To speed up the imputation, we recruited parallel computing with the number of cores set to 8. After obtaining the mediation effect estimates, bias-corrected CIs for the model parameters and mediation effects were constructed. To investigate the nature of the indirect effect, a simple slope analysis was conducted using the *ggeffects* package (version 1.6.0; Comprehensive R Archive Network), estimating marginal means (EMM) for different levels of sleep quality.

## Results

Table 1 displays descriptive results using complete data. Given nonnormality, bivariate correlations were used for gender and IGD. All main variables were positively related to each other, except for age (with no association with IGD and a negative association with executive functioning) and gender (with no association with executive functioning and a negative association with sleep quality). Along with the theoretical support of age and sex effects in IGD [20,84-86], this suggested that gender and age should be regarded as covariates in the next stage of analysis.

Of note, 2.4% (24/1000) of the sample met criteria for IGD, 87.5% (875/1000) who were male, consistent with research in the German adolescent population [7,8]. Additionally, 22.6% (226/1000) of the sample met criteria for chronic sleep reduction according to Youden criteria. Of those who met the criteria for IGD, 54.2% (542/1000) also had chronic sleep reduction. A more detailed description of the demographic characteristics has been reported elsewhere [65].

**Figure 2.**
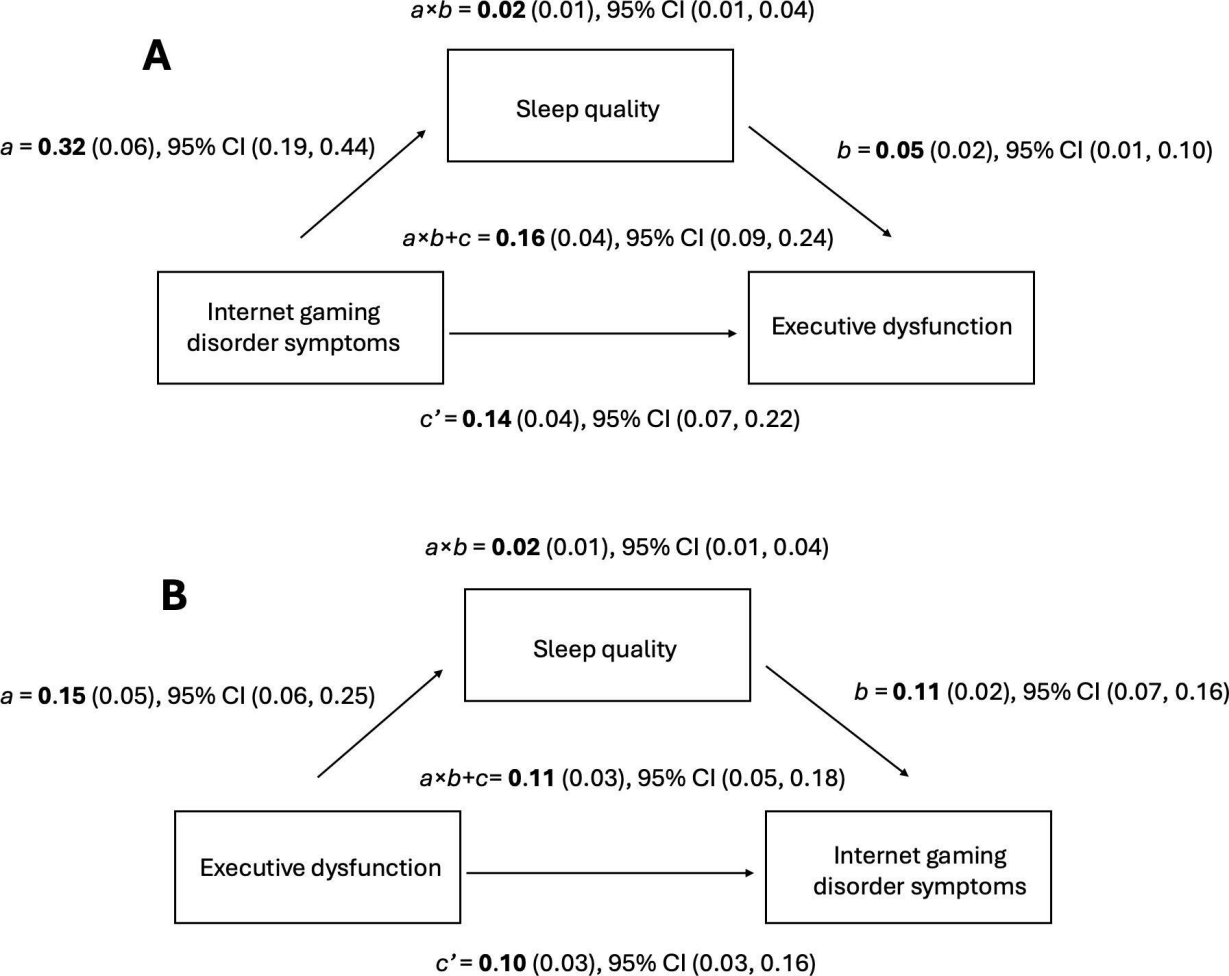
Bootstrap mediation effects of hypothesized models. (A) The model with internet gaming disorder symptoms as the predictor and executive dysfunction as the outcome. (B) The model with executive dysfunction as the predictor and internet gaming disorder symptoms as the outcome. Path coefficients (in boldface) represent parameter estimates, with bootstrap SEs within parentheses. All models were adjusted for age and sex. The 95% CIs were derived from 1000 bootstrap samples, with 60 imputations used to account for missing data. *a* = Internet gaming disorder symptoms’ effect on sleep quality; *b* = Sleep quality’s effect on executive dysfunction; *a*×*b* = The indirect effect; *c*′ = Direct effect, controlling for sleep quality; and *a*×*b*+*c* = Total effect.

First, to evaluate the potential influence of missingness of data, we tested the same models with no imputations. We found negligible differences between the 2 analyses (ie, between missing and complete data). In the first model (Figure 2A), parameter estimates indicated that higher IGD symptoms were associated with poorer sleep quality. Poorer sleep quality was associated with reduced executive functioning. The indirect effects of sleep quality of IGD symptoms on executive functioning were different from zero with 95% CI. Controlling for the indirect effect of sleep quality, IGD remained associated with executive functioning.

In the reverse mediation model (Figure 2B), where executive dysfunction might lead to reduced sleep quality, which in turn could worsen IGD symptoms, the same variables remained significant. Of note, executive dysfunction had a greater association with poor sleep quality. Higher executive dysfunction scores remained associated with IGD symptoms when controlling for the indirect effect of sleep, although this effect was less pronounced compared with the first model. Overall, the results indicated that sleep quality partially mediates the relationship between executive dysfunction and IGD, given that both the indirect and direct effects were significant.

**Figure 3.**
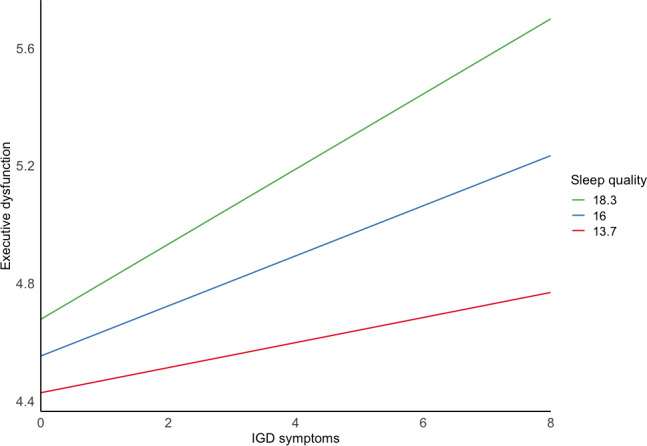
Exploring the indirect effect. Sleep quality categorized as 1 SD above and below the mean, mediating the relationship between IGD symptoms and executive dysfunction. IGD: internet gaming disorder.

To investigate the nature of the indirect effect, a simple slope analysis was conducted. Participants were divided based on sleep quality levels, categorized as 1 SD above and below the mean. As shown in Figure 3, for those with poorer sleep quality (eg, higher scores), IGD symptoms predicted executive dysfunction (EMM 9.13, 95% CI 0.05-0.22). This prediction remained significant for those with average sleep quality, although the effect was reduced (EMM 0.09, 95% CI 0.02-0.17). In contrast, for those with better sleep quality, IGD symptoms no longer predicted executive dysfunction (EMM 0.06, 95% CI −0.05 to 0.17). Thus, the mediating effect of sleep quality between IGD and executive dysfunction diminished as sleep quality improved. Conversely, as sleep quality decreased, its contribution to mediating the relationship between IGD and executive dysfunction increased.

**Table 1.** Descriptives and correlation table.

Variable	Mean/%	SD	Gender (male)	Age	SDQ^a^	IGDS^b^	SRSQ^c^
Gender (male)	53.9% (539/1000)	–^d^	–	–	–	–	–
*r*	–	–	–	0.03	0.04	0.22^e^	−0.03
*P* value	–	–	–	.82	.24	<.001	.001
Age	14.52	1.64	–	–	–	–	–
*r*	–	–	0.03	–	−0.13^e^	0.03	0.15^e^
*P* value	–	–	.82	–	<.001	.21	.001
SDQ^a^	4.65	1.52	–	–	–	–	–
*r*	–	–	0.04	−0.13^e^	–	0.13^e^	0.10^e^
*P* value	–	–	.24	<.001	–	<.001	.001
IGDS^b^	0.86	1.28	–	–	–	–	–
*r*	–	–	0.22^e^	0.03	0.13^e^	–	0.13^e^
*P* value	–	–	<.001	.21	<.001	–	<.001
SRSQ^c^	16.02	2.28	–	–	–	–	–
*r*	–	–	−0.03	0.15^e^	0.10^e^	0.13^e^	–
*P* value	–	–	.001	.001	.001	<.001	–

a SDQ represents the hyperactivity-inattention subscale of the Strengths and Difficulties Questionnaire.

b IGDS measures the severity of symptoms on the Internet Gaming Disorder Scale.

c SRSQ represents the Sleep Reduction Screening Questionnaire.

d Not available.

e Correlation is significant at the .01 level (2-tailed).

## Discussion

### Principal Findings

Research has demonstrated a relationship between IGD and executive dysfunction, indicating that IGD is associated with specific cognitive deficits in attention and hyperactivity or impulsivity. Additionally, both have been linked with poor sleep [58,87-89]. However, the role of sleep as a potential mediator in the relationship between IGD and executive dysfunction remains unclear. We proposed that the current treatment models’ failure to consider sleep as a mediating factor limits their efficacy and overlooks a crucial link in this relationship. Therefore, the aim of this study was to investigate how sleep quality is related to IGD and executive dysfunction. Given the high prevalence of IGD among adolescents and the critical role of executive functioning in their development [15,41,43,85], addressing potential negative impacts remains a key priority.

First, in comparing the strength of the parameter estimates of the 2 models (Figure 3), IGD symptoms appeared to be more strongly linked to sleep quality than executive dysfunction. When controlling for sleep quality, the association between executive dysfunction and IGD was stronger than the reverse. In both models, sleep quality partially mediated the relationship between executive dysfunction and IGD. Partial mediation suggests that while sleep quality accounts for some of the variance in executive dysfunction among individuals with IGD, additional factors—such as reward system dysfunction [90], psychological inflexibility [91], and attentional biases [92]—may also contribute to this relationship. The results of this study indicated that sleep quality may be an important mediatory mechanism when considering the relationship between IGD and executive dysfunction. This is especially relevant, given that more than half of the participants who met criteria for IGD had comorbid chronic sleep reduction, consistent with other research findings [53].

Overall, these findings suggest that the relationship between IGD, executive dysfunction, and sleep quality may operate via a reciprocal feedback loop, with each affecting the other. In other words, IGD can produce poor sleep quality, which in turn can lead to worse executive dysfunction, resulting in a feedback loop. This aligns with previous research demonstrating the reciprocal nature of executive dysfunction and IGD [48,93,94], and, novelly, highlights the role of sleep as a significant mediator.

Categorizing the level of sleep quality allowed us to qualify the nature of its mediation. Specifically, we found that at higher levels of sleep quality, IGD symptoms did not significantly predict executive dysfunction. Conversely, at lower levels of sleep quality, the prediction stood. This suggests that good sleep quality appears to mitigate the impact of IGD on executive dysfunction. Therefore, improving sleep quality may potentially buffer against the negative effects of IGD and executive dysfunction. By extension, identifying poor sleep quality early can help target those at greater risk of compounded symptoms.

### Implications

These findings have several practical and theoretical implications. Previous studies have linked poor sleep status, including reduction in sleep quality, with IGD [53]; however, few current treatment programs include sleep as a factor for improvement [95-98]. Similarly, sleep issues may not always be generally considered in screening procedures. This is despite the evidenced sleep issues among individuals with IGD, including disruptions to the circadian clock [99], increased sleep latency, and decreased total rapid eye movement sleep [100].

From a preventative standpoint, patients presenting with IGD may benefit from a routine assessment of sleep quality during triage, such as the SRSQ or the Sleep Screening Questionnaire Children and Adolescents [101]. Regarding treatment, interventions aimed at improving sleep quality, such as cognitive-behavioral therapy for insomnia, sleep hygiene education, and relaxation techniques, may be particularly beneficial for individuals with IGD and executive dysfunction [102-105]. For instance, programs such as Res@t (Resource-Strengthening Training for Adolescents with Problematic Digital-Media Use) include structured guidance on optimizing sleep routines, such as minimizing screen exposure before bedtime, establishing consistent sleep-wake schedules, and implementing behavioral techniques to reinforce healthier sleep habits [95]. Ensuring adequate and restful sleep may also reduce the risk of developing or exacerbating IGD and executive dysfunction. The bidirectional nature of these relationships underscores the importance of considering multiple factors in management and treatment of IGD.

Ensuring that patients and their families receive psychoeducation regarding the importance of good sleep hygiene and its impact on mental health may help prevent the exacerbation of IGD symptoms and executive dysfunction. Encouraging healthy lifestyle habits that promote better sleep (eg, regular sleep schedules, reduced screen time, physical activity, and blue light filters before bed) can serve as preventive and mitigating measures [106-109]. This can be in addition to psychoeducation regarding the impacts of the sedentary lifestyle, repetitive microtrauma, and obesogenic environment that can accompany excessive gaming and may lead to pathological musculoskeletal injury and a decline in general health status [110].

Finally, impacts on executive functioning are a common finding across addictions. Dysregulated reward processing, diminished impulse control, and aberrant reward-based learning linked to IGD have been similarly found in individuals with gambling disorder [111-114] and substance use disorders [19,44]. Given the partial mediation observed, interventions should consider a multipronged approach that not only addresses sleep hygiene but also directly targets executive function impairments. This ensures that improvements in sleep are supplemented by interventions that target the residual cognitive deficits that persist even when sleep is optimized. Future research could also investigate whether sleep quality, like its role in IGD and executive function, may serve as a potential mediator in other types of addictions.

### Strengths and Limitations

Whereas previous studies investigating IGD are often hindered by small and heterogeneous samples with a lack of focus on possible mediation [26,48], the large sample size in this study is representative of the general population of children and adolescents. Second, the application of bootstrap structural equation modeling to examine mediation, along with multiple imputation to address missing data, follows the latest recommendations for using advanced statistical methods for mediation models [79-82].

Along with its strengths, there are several limitations to consider in this study. The Cronbach α scores for 2 implemented measures (IGDS and SDQ) were lower than usual. As the alpha coefficient reflects both the properties of the scale and the attributes of the sample, unique characteristics in the sample may have contributed to these lower coefficients [115]. For example, it is possible that because the questionnaire was administered over the phone instead of being self-administered, which is the more typical method, respondents might have answered more cautiously to the interviewers in an effort to appear more socially normative or desirable. Supporting this, Jeong et al [116] found a significant discrepancy between self-reported measurements and clinically verified IGD diagnoses among adolescents, with a false-negative rate of 44%. Thus, while the internal consistency of the scales should be interpreted with some caution, it may also be that effects of interest were understated in this sample, and that future studies may find stronger effects. It should also be noted that these 2 scales are widely and successfully used. For example, the IGDS has good established psychometric properties in similar populations [20,117], and the SDQ, one of the most widely used measures of child mental health globally, has been translated into 80 languages [118]. Although the hyperactivity-inattentive subscale on the SDQ has shown promise as a short and efficient screener for executive dysfunction [75,119], future research should also consider using more comprehensive measures such as full neuropsychological batteries that test a range of executive components. Given that ADHD-related symptoms are typically assessed through teacher or parent reports, future studies should incorporate multi-informant assessments. Additionally, future research should also include objective measures of sleep quality, such as actigraphy or polysomnography, instead of relying solely on self-reports, as was the case in this study. The cross-sectional nature of the data limits the ability to infer longitudinal relationships between variables. Therefore, it remains unclear, for instance, whether executive dysfunction leads to the development of IGD or vice versa; only that a relationship exists between these variables. Future longitudinal studies incorporating multimethod sleep assessments will be needed to establish causality. The generalizability of these results is limited to teenagers. Given the vulnerability of children to IGD and the importance of early intervention [49,63,85], it would be beneficial to determine the strength of the tested mediation models in a younger population.

### Conclusions

Recent years have seen a rise in the prevalence of problematic digital media behaviors among adolescents. Executive dysfunction has been associated with IGD, and sleep impairment is also common among adolescents with IGD. The findings of this study align with previous research linking these variables. However, this study’s novelty lies in suggesting that sleep quality can act as a partial mediator between IGD and executive dysfunction. This has significant clinical implications, emphasizing the need to screen for sleep issues as a preventative strategy and to address sleep quality in intervention approaches. Future research is needed to determine the temporal directionality of the relationship between IGD, sleep, and executive functioning incorporating objective sleep measures and comprehensive neuropsychological batteries. Experimental research could also test the effectiveness of sleep-focused interventions in improving cognitive outcomes and reducing IGD symptoms.

## Abbreviations

ADHD: attention-deficit/hyperactivity disorder
*DSM-V-TR*: *Diagnostic and Statistical Manual of Mental Disorders, Fifth Edition, Text Revision*
EMM: estimating marginal means
GD: gaming disorder
*ICD-11*: *International Classification of Diseases, Eleventh Revision*
IGD: internet gaming disorder
IGDS: Internet Gaming Disorder Scale
SDQ: Strengths and Difficulties Questionnaire
SRSQ: Sleep Reduction Screening Questionnaire
